# Athletes' use of analgesics is related to doping attitudes, competitive anxiety, and situational opportunity

**DOI:** 10.3389/fspor.2022.849117

**Published:** 2022-10-18

**Authors:** Marcus Melzer, Anne-Marie Elbe, Katharina Strahler

**Affiliations:** ^1^Department of Sport Psychology, Leipzig University, Leipzig, Germany; ^2^Educational and Training Center, University of Applied Sciences of the Brandenburg Police, Oranienburg, Germany; ^3^Department of Sport and Health Science, Technical University Munich, Munich, Germany

**Keywords:** competitive anxiety, doping attitudes, NSAIDs, situational opportunity, deviance

## Abstract

This study aimed to investigate athletes' hypothetical use of non-steroidal anti-inflammatory drugs (NSAIDs), a behavior similar to doping, and its association with doping attitudes, competitive anxiety and situational opportunity. One hundred twenty-two sport science students completed an online survey assessing biographical information, doping attitudes, and competitive anxiety. Students' intention to use analgesics was measured *via* two different hypothetical situations using the vignette technique. The favorable situation included an absence of potential witnesses and presence of an attractive good whereas witnesses were present in the unfavorable situation and an attractive goal was absent. The results of two hierarchical multiple regression models showed that doping attitudes and competitive anxiety, especially worry, predicted the use of analgesics. In the situation featuring a favorable opportunity, worry was the strongest predictor, whereas in the situation of an unfavorable opportunity, doping attitudes was the strongest predictor for using NSAIDs. Results indicate that NSAID use is associated with positive attitudes toward doping and competitive anxiety, and that it is situationally dependent. Future research perspectives and practical implications are discussed.

## Introduction

“No pain, no gain” is an adage that most athletes would agree with. Elite athletes in particular commonly exceed their physical and psychological limits during training and competition, despite experiencing pain (1, 2). Nixon (3) reported that about 94% of college athletes participated in sports competitions despite being injured. Thus, it appears likely that injured athletes' participation is facilitated by the use of non-steroidal anti-inflammatory drugs (NSAIDs), which are widely used among athletes. Thus, it seems to be obvious that athletes are familiar with the use of painkillers. Studies show that the prevalence of NSAID use in competitive sports ranges from 40 (4) to 86% (5). High rates of NSAID use were identified among Olympic athletes at the Sydney Games (1), in FIFA tournaments (2, 6), as well as in Finnish (7) and Belgian (8) elite athletes. However, the use of NSAIDs is not only a phenomenon related to elite sports. High consumption of NSAIDs has also been identified in non-elite participants of the Bonn (9) and Boston (10) marathons. Athletes consume NSAIDs for the treatment of injury-related pain, pain prevention (prophylactic use), reduction in recovery time and for performance improvement (11–15).

NSAID use is not a violation of Anti-Doping Rules *per se*. Consequently, numerous drugs used to treat pain do not appear on WADA's (16) list of prohibited substances. While NSAID use does not represent an Anti-Doping Rule violation, athletes do mention using them for performance enhancement (13). As a corollary, NSAID use can be seen as a risk factor for the misuse of other substances [see (17, 18)]. In addition, several studies have shown similarities between NSAID use and doping behavior. Research has confirmed that athletes with a strong intention for doping or a history of doping abuse are more likely to misuse other illicit substances [e.g., (19, 20)]. Lippi et al. (21) highlight that there is a “strict parallelism” (p. 105) between doping and medication, nutritional supplements, alcohol or social drug use. The use of NSAIDs has also been related to increased use of doping substances (22). Research shows that medication use is correlated with doping [e.g., (18, 20, 22)]. A question that arises from the many parallels between NSAID use and doping is whether NSAID use, like doping, is also related to positive attitudes toward doping.

Doping behavior is explained as a dynamic interplay between personal attributes (e.g., doping attitudes and/or personality factors) and situational factors (23). Most of the relevant integrative models on doping behavior [e.g., extended Theory of Planned Behavior (TPB), (24), Trans-contextual Model, (25), Life Cycle Model, (26), Sports Drug Control Model (SDCM), (27) describe doping as a goal-directed, intentional and self-regulated behavior that is based on individual decision-making processes both in a broader context and in specific situations. It is, however, unclear whether the use of NSAIDs is determined by a similar interplay between personal and situational factors, and if so, whether certain factors explain NSAID use.

The personality factor competitive anxiety, which has not been widely investigated in relation to NSAID use, deserves special consideration. In fact, few studies focus on the role of anxiety in the use of legal and illegal substances in sports. Studies that have investigated the relationship between anxiety and doping or substance abuse in sport have found a positive correlation [e.g., (28–32)]. Results of these sport-specific studies confirm that one important reason for substance abuse is anxiety reduction (33).

Another aspect relevant to NSAID use is the situation itself. In their doping model, Lazuras et al. (24) include the variable *situational temptation*, which is the perceived temptation to engage in doping under certain conditions. The SDCM (27) also includes situational variables to explain doping, such as drug affordability and availability. Moreover, the SDCM's appraisal of threat and benefit are perceived evaluations of the situational conditions (e.g., perceived likelihood of being tested or sanctioned). According to the SDCM, consumption of doping substances will be high if threat appraisal is low and benefit appraisal is high [see (27)].

Although situation is clearly an explanatory factor in doping behavior models, few studies have investigated effects of the immediate situation on doping use [e.g., (26, 34, 35)]. The Rational Choice Theory [RCT, (36)] postulates that a person is likely to engage in deviant behavior if their assessment of the circumstances leads them to believe they could obtain a desired outcome in which expected gains would outweigh potential costs. According to RCT, a person's chosen methods are then guided and constrained by rational considerations. Following this theoretical viewpoint, individuals seek to maximize personal benefit and minimize costs or risks. Rational decision-making in situations which involve deviant behavior is characterized by a brief cost-and-benefit appraisal. Decisions are predominantly motivated by reward, perceived as temptation, and less by fear of being convicted (37).

Our assumption is that NSAID consumption will be more frequent in low-cost situations in which the benefit is high and risk is low, as opposed to high-cost situations in which the risk of being caught is high and chances for personal benefit are low. Furthermore, we assume that the personality factor anxiety will play a different role in favorable (low-cost) as opposed to unfavorable (high-cost) situations. That is, we assume that anxiety will show a stronger influence on NSAID use in favorable situations characterized by low costs (38).

On the other hand, a positive attitude toward doping is assumed to have a strong impact on doping behavior in both favorable and unfavorable situations. This is based on results showing that perceived opportunity does not substantially influence behavior when strong attitudes prevail [see (39)]. In a way similar to a distorting lens, strong attitudes affect perception of a situation and lead to less deliberation and an automatic behavior pattern regardless of situational context factors. Once in a situation, perceptive biases lead to a strong and easily assessable doping attitude, which may in result in positive doping behavior, and/or the decision to use NSAIDs.

## Research questions

This study investigates three research questions. The first question interrogates whether the use of NSAIDs, a behavior presumably analogous to doping, is related to doping attitudes. We assume positive correlations between the hypothetical consumption of NSAIDs and doping attitudes.

The second research question investigates if competitive anxiety is related to situations characterizing NSAID use. We assume that competitive anxiety is related to the hypothetical use of NSAIDs.

The third question investigates if the likelihood of NSAID use is dependent on situational condition. We assume that NSAID use is indicated if the situation is favorable rather than unfavorable. A favorable situation is characterized by the absence of potential witnesses and the presence of an attractive good (40). We also assume that anxiety is more strongly related to NSAID consumption if the situation is favorable rather than unfavorable.

## Materials and methods

### Participants and procedure

Study participants were recruited by means of different sport-related online social media groups as well as through the university mailing list. Participants completed an online version of the questionnaire that was programmed using EFS Survey 6.0 from Globalpark (41). A total of 198 participants completed the survey. However, 76 responses were deleted due to too many missing items and apparent inconsistencies in the data set. Some participants could not be categorized as athletes due to missing values in variables like sports, competition experience and national squad membership. The final sample of *N* = 122 male and female athletes (50% male) had a mean age of *M* = 24.98 ± 4.52 years. Approximately 62% of participants took part regularly in sport competitions, with an average number of annual competitions of *M* = 15 ± 12. Competitions ranged from national competitions to international championships. About a quarter (27%) of participants were members of a national team. Nearly 56% of athletes participated in team sports (predominantly football, volleyball or handball) while the remaining participants took part in individual sports (predominantly track and field, swimming or cycling).

### Instruments

#### Biographical information

Participants provided demographic information about their age, gender, athletic status, number of yearly competitions and sport league status.

#### Doping attitudes

The German version of the Performance Enhancement Attitude Scale [PEAS, (42)] was used to measure doping attitudes. The PEAS consists of 17 items (e.g., “Doping is necessary to be competitive”) rated on a 6-point Likert rating scale ranging from 1 = “strongly disagree” to 6 “totally agree.” The internal consistency (Cronbach's Alpha) of α = 0.81 was comparable to the one reported in Folkerts et al.'s (43) systematic review.

#### Analgesic use intention

The intention to use NSAIDs was measured *via* two scenarios (40). While one scenario presented a favorable situation, the other presented an unfavorable situation in which the use of NSAIDs was set. Participants were asked to rate the likelihood of taking NSAIDs on a 7-point Likert-scale ranging from 0 = “very unlikely” to 6 = “very likely.”

The favorable situation was defined by the presence of an attractive good (e.g., glory) and the absence of witnesses.

##### Favorable situation (low cost)

*Imagine the following situation*. A young professional football player wants to play in the final point game, the city derby, which is the highlight of the season despite the pain he has suffered due to a previous injury. When warming up, his thigh hurts and he has problems running quickly. In order to participate, he needs to take painkillers. He goes back to the empty locker room where he takes several painkillers in the bathroom.

In contrast, the unfavorable situation was characterized by the presence of witnesses and the absence of an attractive good.

##### Unfavorable situation (high cost)

*Imagine the following situation*. A young professional football player wants to play in his team's first pre-season test match despite experiencing pain due to a previous injury. When warming up, his thigh hurts and he has problems running quickly. To participate, he needs to take painkillers. Before the game starts, he pulls out the painkillers in the locker room where his teammates are getting changed and he goes to the bathroom to drink some water.

#### Competitive anxiety

Competitive anxiety was measured with the German Sport Anxiety Scale [WAI-T: Wettkampfangstinventar, (44)]. The WAI-T assesses three dimensions of competitive anxiety–worry (e.g., “Before a competition, I am worried about choking under pressure.”), somatic anxiety (e.g., “Before a competition, my heart is racing.”) and concentration disruption (e.g., “Before a competition, it is hard for me to keep my thoughts focused on the competition.”). Respondents rated the 12 items on the questionnaire according to a 4-point Likert scale, ranging from 1 = “not at all” to 4 = “very.” Internal consistency (Cronbach alpha) of somatic anxiety (α = 0.88) and worry (α = 0.82) were good. However, Cronbach's alpha for concentration disruption (α = 0.54) was too low. Therefore, this scale was excluded from further analyses.

#### Data analysis

Data were analyzed using SPSS Version 17. Statistical analyses included bivariate Pearson correlations, a univariate repeated measures ANOVA, and hierarchical multiple regression modeling. The level of significance was fixed at *p* < 0.05. In addition to significance testing, we reported a “two-step-process” with relevant effect sizes and statistical power (1-β) in both hierarchical linear regression models (45). G^*^Power 3.1.9.2 was used for our analyses.

## Results

Descriptive analysis results are presented in Table 1. Correlations between the measured dependent and independent variables are presented in Table 2.

**Table 1:** Mean, SD, min, max and range of independent, and dependent variables.

**Variable**	**M**	**SD**	**Min**	**Max**	**Range**
1. Doping attitudes	32.13	10.20	1	5	4
2. Somatic anxiety	10.84	2.88	4	16	12
3. Worry	10.11	2.73	4	16	12
4. Concentration disruption	7.09	2.03	4	13	9
5. Analgesic misuse in a favorable situation	2.96	1.74	1	6	5
6. Analgesic misuse in an unfavorable situation	2.16	1.42	1	6	5

M, mean; SD, standard deviation; Min, minimum; Max, maximum; Range, range of data set.

**Table 2:** Bivariate Pearson correlations of the predictors and outcome variables.

**Variable**	**1**	**2**	**3**	**4**	**5**
1. Analgesic misuse-favorable situation	-				
2. Analgesic misuse-unfavorable situation	0.62^**^	-			
3. Doping attitudes	0.26^*^	0.35^**^	-		
4. Worry	0.33^**^	0.22^*^	−0.02	-	
5. Somatic anxiety	0.18^*^	0.09	0.08	0.51^**^	-

* *p* < 0.05,

** *p* < 0.01.

First, we analyzed if doping attitudes were related to NSAID use. Bivariate correlation analyses showed positive significant correlations for both unfavorable and favorable NSAID use conditions.

In a second step, we investigated correlations between the two situations and anxiety. We found positive correlations between the favorable condition and worry and somatic anxiety, and between the unfavorable situation and the worry component of competitive anxiety.

In a third step, we investigated the difference between NSAID use in favorable and unfavorable situations with a univariate repeated measures ANOVA. The mean difference was significant and the effect size was substantial *F*_(1, 121)_ = 38.90, *p* < 0.01, η^2^= 0.24. Athletes were more likely to use NSAIDs in the favorable than in the unfavorable situation.

Lastly, we investigated the influence of doping attitudes and competitive anxiety on NSAID use in both situations. Prior to conducting the two hierarchical regression analyses, we verified assumptions for regression analysis and considered biasing influences (46). These tests confirmed satisfactory quality of the data set for conducting a hierarchical regression analysis.

### Hierarchical regression model for NSAID misuse in a favorable situation

We started with the hierarchical regression model for the dependent variable “analgesic misuse in a favorable situation.” In a first block, we added age and gender as possible covariates and participation in competition to control their impact on decision-making [see (47, 48)]. The model did not significantly predict the dependent variable, *F*_change_ <1, *p* = 0.51. The three variables showed no significant influence on decision-making in a favorable situation (see Table 3). *Post-hoc* power analysis indicated a poor statistical power of *1–*β = 0.22, which clearly missed the threshold of 0.80 [see (49)].

**Table 3:** Hierarchical regression model of analgesic misuse in a favorable situation.

**Variable**	***R^2^***	***R^2^*** **Change**	**B**	**SE B**	**β**	***t***
Step 1	0.02					
Constant			2.13	0.99		2.16
Age			0.02	0.04	0.05	0.54
Gender			0.05	0.32	0.01	0.14
Competition participation			0.50	0.34	0.14	1.47
Step 2	0.09^*^	0.07^**^				
Constant			1.03	1.02		1.01
Age			0.01	0.04	0.02	0.22
Gender			−0.19	0.32	−0.06	0.55
Competition participation			0.50	0.33	0.14	1.53
Doping attitudes			0.80	0.27	0.28^**^	3.01
Step 3	0.18^**^	0.09^**^				
Constant			−0.57	1.07		−0.53
Age			>-0.01	0.03	−0.01	−0.10
Gender			0.04	0.31	0.01	0.12
Competition participation			0.50	0.31	0.13	1.44
Doping attitudes			0.70	0.27	0.24^**^	2.72
Worry			0.20	0.06	0.31^**^	3.60

* p < 0.05,

** p < 0.01. R^2^, Proportion of variation in dependent variable; R^2^ Change, Proportion of variation in dependent variable by independent variable; B, Unstandardized regression coefficient; SE B, Standard deviation of unstandardized regression coefficient; β, Standardized regression coefficient; t, T-statistic for regression coefficient.

We then introduced doping attitude to the model. The second model showed a significant improvement in prediction, *F*_change(1, 117)_ = 9.05, *p* < 0.01, and the explained variance increased to 9%. A *post-hoc* power analysis of the change between the first and the second step with a nominal α = 0.05 and the partial *R*^2^ = 0.07 showed a statistical power of *1–*β = 0.85 which exceeded the convention of 0.80 and represented a substantial gain.

In the last block, we included the two components of competitive anxiety (worry and somatic anxiety) stepwise to the model. Only worry had a significant impact; and the regression model explained an additional 9% of the variation (*F*__*change*_(1, 117)_ = 12.98, *p* < 0.01). Relevant predictors in the regression model were doping attitudes and the worry component of competitive anxiety. Adjusted explained variance was 15%. The *post-hoc* power analysis of the change between step two and three revealed a satisfying power of *1–*β = 0.93.

### Hierarchical regression model for NSAID misuse in an unfavorable situation

Following the analysis of NSAID misuse in a favorable situation, we proceeded in the same way for NSAID misuse in an unfavorable situation (see Table 4).

**Table 4:** Hierarchical regression model of analgesic misuse in an unfavorable situation.

**Variable**	***R^2^***	***R^2^*** **Change**	**B**	**SE B**	**β**	***t***
Step 1	0.03					
Constant			2.43	0.80		3.02
Age			−0.02	0.03	−0.06	−0.65
Gender			−0.05	0.26	−0.02	−0.20
Competition participation			0.40	0.28	0.14	1.45
Step 2	0.17^**^	0.15^**^				
Constant			1.14	0.80		1.43
Age			−0.03	0.03	−0.11	−1.21
Gender			−0.33	0.25	−0.12	−1.34
Competition participation			0.40	0.25	0.14	1.59
Doping attitudes			0.94	0.21	0.40^**^	4.53
Step 3	0.20^**^	0.03^**^				
Constant			0.37	0.86		0.43
Age			−0.04	0.03	−0.12	−1.42
Gender			−0.22	0.25	−0.08	−0.89
Competition participation			0.38	0.25	0.13	1.51
Doping attitudes			0.89	0.21	0.38^**^	4.32
Worry			0.10	0.04	0.18^*^	2.15

* p < 0.05,

** p < 0.01. R^2^, Proportion of variation in dependent variable; R^2^ Change, Proportion of variation in dependent variable by independent variable; B, Unstandardized regression coefficient; SE B, Standard deviation of unstandardized regression coefficient; β, Standardized regression coefficient; t, T-statistic for regression coefficient.

The regression model with age, gender and competition participation showed no significant influence on the independent variable (*F*_change(3, 118)_ = 1.09, *p* = 0.36). The statistical power of the model was also poor, *1–*β = 0.33. Adding doping attitudes (*F*__*change*_(1, 117)_ = 20.55, *p* < 0.01) to the model increased explained variance up to 17%. A *post-hoc* power analysis showed a perfect power.

In the last step, we also introduced competitive anxiety to the model in a stepwise manner. The competitive anxiety scale “worry” significantly explained 3% variation of NSAID misuse, *F*_change(1, 116)_ = 4.60, *p* < 0.05. The total regression model of NSAID misuse in an unfavorable situation explained the adjusted variance of 17%. The power of adding the last predictor was made according to the convention of 0.80.

## Discussion

With regard to the first set of research questions, results indicate that the decision to use NSAIDs is moderately correlated with doping attitude. This supports the assumption that the use of NSAIDs is a behavior parallel to doping. This result confirms previous research indicating that doping behavior and NSAID consumption are correlated (22).

With regard to the second research question investigating competitive anxiety, results show that the worry component in particular is associated with NSAID use in both situational conditions. Athletes with a tendency to experience high worry and high somatic anxiety in competitions are more likely to use analgesic substances in favorable situations. Athletes with a tendency to experience only high worry in competitive situations are even more likely to use analgesic substances regardless of the situation. So far, the relationship between anxiety and NSAID use has not been widely investigated. The results of this study contribute to furthering our understanding of the relationship between the two and suggest that NSAID consumption could be related to anxiety reduction (33).

With regard to the third research question, our study results indicate that the immediate situation is relevant for NSAID use. The likelihood of using NSAIDs depends on situational conditions. Our study confirms that athletes were more likely to consume NSAIDs in a favorable situation with a lower risk of detection and expected higher benefits. These results are in line with the Low-Cost Hypothesis [for an overview: (38)] and Rational Choice Theory (36). Previous studies of deviant and criminal behavior have shown that if the perceived benefit exceeds the expected risk, the likelihood of different deviant behaviors such as shoplifting, drunk driving, stealing or buying illegal drugs increases, as well [e.g., (40, 50–52)]. Our results are also in line with the expected outcome of the SDCM (27) and the Drugs in Sport Deterrence Model [DSMD, (53)].

We also assumed that anxiety is more strongly related to NSAID consumption if the situation is favorable rather than unfavorable. Looking at the correlations we can see that worry and somatic anxiety were positively related in the favorable situation, whereas only worry was correlated in the unfavorable situation. Results of the regression analysis highlight that in the favorable situation, worry had a significant impact on NSAID use, which explained even more variance than doping attitudes. In the unfavorable situation, worry was also a significant predictor; however, doping attitudes explained significantly more variance regarding impacts on NSAID use. This means that in a situation with little risk of being caught and some incentive, athletes with high somatic anxiety and high worry might cope with the stressful situation by resorting to substance use. In the unfavorable situation, however, substance use among athletes experiencing higher levels of worry is more dependent on whether they also have positive attitudes toward doping and not solely explained by their high worry. These results confirm our hypothesis that the relationship between anxiety and NSAID consumption depends on perceived cost of the situation, whereby anxiety seems to play a larger role if situational costs are low.

Our results suggest that factors contributing to NSAID consumption are associated to those of doping, as is the case with analogous behaviors such as nutritional supplement intake or recreational and illicit drug use (18, 19, 54, 55). Therefore, NSAID use should be considered a gateway for doping, as both share similar underlying mechanisms (18, 55). Furthermore, our results indicate that not only athletes with positive doping attitudes but also athletes with high anxiety are at risk, especially in the favorable situation.

### Limitations and future research perspectives

Some limitations need to be discussed with regard to our study. First, we assessed only one situation for consumption of NSAIDs. Although this situation had been extensively pre-tested, future studies should include a variety of situations. Second, this study assessed doping attitudes with an explicit measure. Future studies could assess doping intentions using implicit measures to capture more spontaneous reactions rather than replies based on deliberation (56). Due to the exploratory character we did not include a power analysis beforehand. We did, however, use G-Power for *post-hoc* power analysis of effect sizes for the included predictors in the hierarchical regression analysis. Future studies should include a priori power analysis for optimal sample size. In addition, we only investigated two personality factors, namely competitive anxiety and doping attitudes. Additional personality factors of relevance should be identified and included in future studies. Ring et al. (57), for example, mention the importance of self-enhancement values, which could be implicated in future research on the impact of situation on the decision to engage in substance consumption. Self-enhancement values can lead to stable and chronic attitudes (e.g., doping attitudes) which impact decision-making. A further limitation is that only two of the three subscales of the competitive anxiety scale could be used due to insufficient reliability.

### Practical implications

Our study results have several practical implications especially with regard to doping research and doping prevention. Results indicate that NSAID consumption can be characterized as a behavior analogous to doping and should be considered as a gateway to doping, similar to nutritional supplementation. Accordingly, future studies could employ NSAID consumption as a behavior analogous to doping. While athletes might not be keen to reply to questions involving actual doping behavior, they might be more open to answering questions about NSAID use. Furthermore, our study shows that favorable situations–with expected high glory and low chance of detection–facilitate deviant behavior. Our study results could therefore be used to further specify the elements of the SDCM model by adding additional information about situational factors (e.g., favorable/unfavorable situation). With regard to prevention, our results suggest that situations in which athletes have easy and unsupervised access to substances should be avoided. Furthermore, awareness should be raised that athletes with positive doping attitudes are at risk to consume substances in both unfavorable and favorable situations. It is possible that athletes reflect on their decisions in unfavorable situations and that their decisions to take the substances are not automatic. Thus, training of ethical decision-making is indicated as a feasible approach to prevention (58, 59). Finally, and importantly, prevention efforts should consider focusing on reducing anxiety as anxiety has been shown to be correlated with NSAID consumption.

## Conclusion

This study identified that NSAID use can be described as a behavior analogous to doping. Furthermore, the personality factor competitive anxiety, in addition to doping attitudes, was found to be related to NSAID consumption. Therefore, further research should examine whether competitive anxiety is a potential risk factor for doping. The study further highlights that conducting research on doping should consider the situation. That is, different factors influence the decision for NSAID use in unfavorable vs. favorable situations.

## Data availability statement

The raw data supporting the conclusions of this article will be made available by the authors, without undue reservation.

## Ethics statement

Ethical review and approval was not required for the study on human participants in accordance with the local legislation and institutional requirements. The patients/participants provided their written informed consent to participate in this study.

## Author contributions

MM designed the study and collected and analyzed the data. KS and MM drafted the manuscript. KS and A-ME conducted additional data analyses. A-ME critically reviewed the manuscript. All authors contributed to the article and approved the final version.

## Conflict of interest

The authors declare that the research was conducted in the absence of any commercial or financial relationships that could be construed as a potential conflict of interest.

## Publisher's note

All claims expressed in this article are solely those of the authors and do not necessarily represent those of their affiliated organizations, or those of the publisher, the editors and the reviewers. Any product that may be evaluated in this article, or claim that may be made by its manufacturer, is not guaranteed or endorsed by the publisher.
